# The Role of Diet in Multiple Sclerosis: Mechanistic Connections and Current Evidence

**DOI:** 10.1007/s13668-018-0236-z

**Published:** 2018-08-16

**Authors:** Ilana Katz Sand

**Affiliations:** Corinne Goldsmith Dickinson Center for Multiple Sclerosis, Department of Neurology, Icahn School of Medicine at Mount Sinai, 5 East 98th Street, Suite 1138, New York, NY 10029 USA

**Keywords:** Multiple sclerosis, Diet, Nutrition, Experimental autoimmune encephalomyelitis, Mechanism, Environment

## Abstract

**Purpose of Review:**

This review seeks to examine current research related to the role of diet in multiple sclerosis (MS).

**Recent Findings:**

Recent research in preclinical models, epidemiologic studies, and limited prospectively followed cohorts provide preliminary evidence that dietary factors influence MS incidence, disease course, and symptomatology. Current evidence for the effects of fatty acids, fruits and vegetables, whole grains, dairy, and salt are reviewed. Dietary patterns including overall diet quality, caloric restriction, McDougall diet, Paleolithic diet, and Mediterranean diet are discussed. Hypotheses regarding potential mechanistic connections underlying observed effects are also presented.

**Summary:**

Several individual dietary components and patterns demonstrate potential for significant impact in MS. Definitive answers regarding the ability of diet to act as a disease modifier in MS will ultimately require large-scale clinical trials. Continued prospective studies and clinical trials to further advance this line of research are warranted.

## Introduction

Multiple sclerosis (MS) is a chronic central nervous system disease with inflammatory and neurodegenerative components. Most patients initially present with a relapsing-remitting course defined by the acute onset of focal neurologic deficits and corresponding focal inflammatory changes visible on MRI. Episodes reflect inflammatory demyelinating lesions in the optic nerves, brain, and spinal cord that result in symptoms such as loss of vision, weakness, numbness, gait difficulty, and bowel and bladder disturbances [1]. Many MS patients also experience more global symptoms such as fatigue, depression, and cognitive changes. In addition to inflammatory lesion formation, atrophy indicative of neurodegeneration begins early in the disease course [2] and drives disability over time [3]. Approximately 10% of MS patients demonstrate disease progression characterized by insidious neurologic decline from the outset (primary progressive MS) while a much larger subset begins with a relapsing-remitting course but develops symptoms of gradual decline later on (secondary progressive MS) reflective of worsening neurodegeneration even in the absence of new inflammatory lesions [4].

MS is thought to result from a combination of genetic predisposition and environmental influences [5, 6]. Established environmental risk factors for MS include low vitamin D levels [7], sun exposure [8], smoking [9], and viral exposures [10]. Relevant to this review, obesity, particularly during adolescence, has also been identified as a risk factor for MS [11]. In addition to the importance of environmental factors in risk of disease onset, there is significant variability among MS patients regarding clinical disease course. This observation has resulted in a search for modifiable environmental factors that might be manipulated to positively impact outcomes once the diagnosis of MS has been established. Many of the above risk factors for MS development have also been demonstrated to impact clinical course [12–16]. However, a significant portion of environmental risk for the development of MS as well as variability in the clinical MS disease course currently remains unexplained. Epidemiologic research dating back many years has suggested dietary factors may be important in MS [17]. While large-scale clinical trial data is not yet available, a growing body of literature including epidemiologic, preclinical, and observational studies as well as small clinical trials suggests the importance of dietary factors in the risk of MS onset and clinical course. The diet is a major source of environmental interaction and dietary metabolites exert far-reaching systemic effects, rendering diet an attractive candidate as a potential environmental mediator in MS. This review will begin with a discussion of potential underlying mechanisms for the effects of diet as a disease modifier followed by presentation of the current literature on various dietary factors in MS. Potential effects of diet on MS symptoms will also be discussed.

## Multiple Sclerosis Pathophysiology: Opportunities for Dietary Effects

There are at least three overarching aspects of MS pathophysiology that represent opportunities for influence on disease outcomes. Interventions may (1) modulate the inflammatory state, (2) protect against neurodegeneration, or (3) promote nervous system repair.

The pathophysiology of MS is complex and incompletely understood [18•]. Supporting the hypothesis that MS is at least in part related to peripheral autoimmunity, genome-wide association studies have mapped MS risk to loci related to the immune system [19, 20]. MS was once considered strictly related to T cell dysregulation; however, many components of the innate and adaptive immune systems have now been shown to be relevant to MS immunopathology [18•, 21]. The complexity of MS pathophysiology is reflected by the myriad therapies approved for relapsing MS, each with a distinct immunomodulatory mechanism yet all effective at preventing clinical disease relapses and new lesions on MRI. Dietary factors that are able to promote regulatory as opposed to inflammatory immune cell differentiation and cytokine production therefore have the potential ability to reduce new inflammatory lesion formation and clinical relapses utilizing pathways similar to traditional disease-modifying therapies for MS.

Current MS disease-modifying therapies are relatively effective at reducing new lesions and clinical relapses; however, more elusive have been agents that halt underlying neurodegeneration. As described, approximately 10% of patients present with progressive neurological decline from the outset. Even in patients with a relapsing-remitting phenotype, the neurodegenerative aspect of the disease is present early on and is in fact detectable before the disease becomes clinically apparent [22]. Histology in chronic MS demonstrates evidence of smoldering localized inflammation, chronic demyelination, and axonal and neuronal damage at the site of MS lesions as well as more broadly, affecting both white matter and gray matter [23•]. According to a model with increasing support, oxidative stress causes mitochondrial dysfunction resulting in chronic energy insufficiency, eventually leading to ion channel redistribution causing cell damage and eventually cell death [24]. Effective disease-modifying therapies in relapsing patients slow but do not halt this neurodegenerative process. There is therefore a great need for the identification of strategies that are able to protect against chronic demyelination and axonal/neuronal loss. Dietary factors that dampen resident CNS inflammation, combat oxidative stress, or protect mitochondria may help prevent chronic demyelination and axonal/neuronal damage.

Also currently under study are multiple strategies aimed at remyelination and repair. Remyelination of demyelinated axons may be helpful with regard to restoring function as well as with protection of previously denuded axons from further damage. The process of remyelination occurs spontaneously however is highly variable between individuals and efficiency decreases over time [25]. Oligodendrocyte precursor cells (OPCs) capable of remyelination are present in the adult brain; however, inhibitors of OPC differentiation in the local environment hinder this process [26]. Therapies aimed at manipulating the CNS environment to favor OPC differentiation and encourage remyelination are currently in clinical trials [27]. Dietary factors that are able to influence remyelination and repair could certainly be of benefit in MS.

## Mechanisms for Dietary Effects in Multiple Sclerosis

There are several theoretical pathways through which dietary factors may exert systemic influence resulting in beneficial effects on inflammation, neuroprotection, and repair in MS. The first is through indirect effects on metabolic factors. Diet has a significant impact on body weight, cholesterol levels, and other vascular risk factors that affect MS risk and disease course [11, 28–33]. Separating the relative contribution of diet from these other factors is challenging and will require targeted studies. A detailed discussion of the role of these metabolic factors in MS has been completed elsewhere [34, 35]; this review will focus on direct effects of diet in MS. These include effects mediated by dietary metabolites derived directly from food, dietary induction of metabolite production by gut microbiota, and diet-mediated changes in gut microbial composition.

Whether metabolites arise directly from the diet or are produced by gut bacteria in response to ingestion of particular foods, they exert effects through shared mechanisms. These include G-protein coupled receptor (GPCR) signaling as well as epigenetic regulation of gene expression through inhibition of histone deacetylases (HDAC) and effects on transcription factors such as the aryl hydrocarbon receptor (AhR) [36]. In addition to their presence on intestinal epithelium and tissues relevant to metabolism such as adipose tissue, “metabolite-sensing” GPCRs are expressed by various immune cells. Potentially important for observed effects in MS, for example, GPR120 recognizes omega-3 fatty acids [37], GPR35 recognizes tryptophan metabolites [38], and GPR43 recognizes short-chain fatty acids (SCFAs) [39]. Interestingly, one signaling pathway engaged by many GPCRs is mediated by β-arrestin-2, resulting in downstream inhibition of NF-κB and other anti-inflammatory effects [36]. Regarding epigenetic regulation, acetylation of histones mediated by HDAC inhibitors largely results in transcriptional activation. Relevant to MS, HDAC inhibitors have generally been demonstrated to have anti-inflammatory effects including increased Foxp3 expression and increased numbers of Foxp3+ Treg cells [40]. Butyrate and the other SCFAs are natural HDAC inhibitors; other dietary metabolites may act through these pathways as well. The aryl hydrocarbon receptor (AhR) is a ligand-activated transcription factor stimulated by a broad array of small molecules including many that arise from environmental exposures. AhR activation is associated with a variety of anti-inflammatory effects on the innate and adaptive immune systems [41]. Particularly pertinent for MS, dietary-derived metabolites are able to cross the blood-brain barrier and activate AhR on astrocytes resulting in targeted, local effects in the CNS [42, 43••]. The most well-characterized AhR agonists relevant to MS are tryptophan and its metabolites. Flavonoids are also able to act as AhR agonists (both discussed further below).

Some of these metabolites are derived directly from the diet whereas others are produced by resident commensal flora, the gut microbiota. Studies in experimental autoimmune encephalomyelitis (EAE; MS animal model) and in MS demonstrate important links to gut microbial composition [44••, 45, 46, 47•, 48–50] and suggest manipulation of microbiota by diet may be of benefit [51]. Dietary factors induce the production of particular metabolites by gut microbiota as well as more indirectly affect metabolite production by affecting gut microbial composition. In addition to effects mediated by metabolites, gut microbiota directly interact with the gut’s resident immune system. Pattern recognition receptors (e.g., toll-like receptors) on intestinal epithelial and dendritic cells recognize microorganism-associated molecular patterns and subsequently influence T cell differentiation in mesenteric lymph nodes through effects on antigen presentation [52, 53]. As different microorganisms induce diverse effects, influences on microbial composition are significant determinants of systemic impact. Many factors affect gut microbial composition including mode of birth and breastfeeding [54], infectious and antibiotic exposure [55, 56]; however, the biggest long-term driver is diet [57•, 58], thereby providing an important potential mechanistic link in MS.

## Evidence for the Role of Specific Dietary Components in Multiple Sclerosis

### Fats

#### Saturated Fat

Saturated fats, defined by the presence of the maximum number of hydrogen atoms bound to each carbon atom in the fatty acid chain, have been linked to inflammation and largely blamed for the rise in cardiovascular disease in developed countries [59]. Several mechanisms are potentially relevant to MS. Though recent questions have been raised, it is generally accepted that intake of saturated fats increases LDL cholesterol [59], which is associated with poor outcomes in MS [30–33]. Saturated fats directly impact the innate immune system through activation of pro-inflammatory toll-like receptors, leading to downstream consequences including increased NF-κB [60]. They may also indirectly affect these pathways by increasing endotoxin levels [61]. Effects on the adaptive immune system are also likely important. In an animal model of MS, mice fed a “Western” high fat diet exhibited worsened clinical scores associated with increased T cell and macrophage infiltration and spinal cord expression of pro-inflammatory cytokines IL-1B, IL-6, and IFNγ [62]. Interestingly, fatty acid chain length seems to be an important determinant of ultimate effects. Long-chain fatty acids (LCFA) such as those commonly found in processed foods featured in “Western” diets promote the differentiation of naïve T cells into pro-inflammatory T_H_1 and T_H_17 cells, seemingly mediated by members of the MAP kinase family including p38 and JNK1 [63]. Mice fed an LCFA-rich diet exhibited more severe EAE compared to those fed a standard diet. In contrast, short-chain fatty acids (SCFAs) have been demonstrated to favor differentiation of regulatory T cells with resulting production of anti-inflammatory cytokines [63, 64]. Rather than being ingested in the diet, SCFAs are largely produced by intestinal microbiota in response to dietary intake of fiber-rich plant-based foods and are therefore discussed below (see “Fruits, Vegetables, and Whole Grains”).

From an epidemiologic standpoint, Swank observed geographic variations in MS incidence that could be related to intake of fats, particularly fats derived from animal products such as meat and dairy, first published in 1950 [17, 65]. He subsequently led a prolonged study that began in 1951 when 144 MS patients agreed to follow a diet with reduced saturated fat (< 20 g/day) intake. Participants were later classified as “good dieters” if they continued following this recommendation or “poor dieters” if they consumed more than 20 g of saturated fats per day. Clinical follow-up continued for many subjects through 34 years [66•] and for a smaller group through 50 years [67]. Those who adhered to the diet showed significantly less disability and had lower mortality rates than those who did not. However, lack of randomization or measurement of potential confounding factors makes these results difficult to interpret. Still, these are interesting observations that warrant follow-up in the modern research era.

More recently, a prospective study in pediatric MS with rigorously collected disease information from 219 participants followed for a median of nearly 2 years noted an association between energy intake from fat and relapse rate (adjusted hazard ratio for 10% increase in energy intake from fat 1.56, *p* = 0.027) [68••]. Notably, the same increase in energy intake from saturated fat *tripled* this risk (adjusted hazard ratio for 10% increase in energy intake from saturated fat 3.37, *p* = 0.009). These associations remained after adjustment for potential confounders including age, gender, ethnicity, socioeconomic status, disease duration, use of MS disease-modifying therapy, total energy intake, and BMI.

To date, there have been no prospective controlled trials focusing on saturated fat intake, though the McDougall diet, a very low-fat, plant-based diet (discussed below) is currently being investigated as a potential strategy to manage fatigue in MS.

#### Polyunsaturated and Monounsaturated Fats

Polyunsaturated fatty acids (PUFAs) contain multiple double bonds within the fatty acid chain and are found in foods such as fish, walnuts, and flax seeds. In particular, there is high interest in omega-3 fatty acids (first double bond at the third carbon position), especially the largely plant-derived alpha-linolenic acid (ALA) and largely marine-derived eicosapentaenoic acid (EPA) and docosahexaenoic acid (DHA). PUFAs decrease inflammation through conversion into the anti-inflammatory prostaglandins E1 and E2, with downstream effects on cytokine production, leukocyte migration, and other immune system components [69]. Animal studies have demonstrated beneficial effects of PUFAs on EAE including decreased production of inflammatory cytokines and induced peroxisome proliferator-activated receptors (PPAR) in CNS infiltrating T cells [70, 71]. In addition to immunomodulatory effects, animal models suggest PUFAs prevent demyelination and promote neuroprotection and remyelination. In a toxic demyelination animal model of MS (cuprizone), pretreatment of rats with EPA resulted in decreased weight loss and increased whole brain cerebroside content [72]. Mice treated with cuprizone and fed a salmon diet showed smaller lesion volume, less demyelination, and enhanced remyelination in the corpus callosum compared to controls [73].

Epidemiologic studies relating to PUFA intake in MS patients have shown conflicting results. A prior study utilizing the Nurses’ Health Study I and II cohorts that identified 195 incident cases of MS among nearly 200,000 enrollees noted a non-significant trend toward an inverse relationship between ALA intake and MS incidence [74]. A follow-up study with extended observation period identified 479 incident MS cases and did note a significant inverse association between both total PUFA intake and ALA and MS risk (HR top vs. bottom quintile 0.67 and 0.61, respectively) [75]. However, other studies suggest the importance of marine-based PUFA intake. A Swedish case-control study found a decreased incidence of MS among those who reported high fatty fish intake (adjusted odds ratio 0.82) [76]. Similarly, an Australian study found significantly decreased risk of a first clinical demyelinating event among those who reported high intake of omega-3 fatty acids (adjusted odds ratio 0.61) especially when these were marine rather than plant derived (adjusted odds ratio 0.54) [77]. An additional study noted a link between fresh fish intake and onset of MS, independent of vitamin D levels [78].

Clinical trials regarding PUFA intake have yielded inconsistent results [79–83]. Notably, trials conducted thus far have largely been related to PUFA supplements rather than foods though some have also included dietary recommendations such as a study of 31 MS patients randomly assigned to supplement a low-fat diet with fish oil vs. olive oil supplement [83].

Monounsaturated fats (MUFAs), with a single double bond in the fatty acid chain, are found in foods such as olive oil, avocados, and certain nuts. To date, there has been little experimental work directly related to monounsaturated fatty acid intake and MS onset or disease course. However, they are of potential interest in MS given the established benefits of Mediterranean-style diet in cognitive aging [84, 85•] given parallels with MS as a neurodegenerative disease. A pilot clinical trial of a modified Mediterranean diet high in PUFAs and MUFAs (see below) is currently underway.

### Dairy

Vitamin D deficiency is a risk factor for the development of MS [7, 86] and also a negative prognostic factor in MS [87]. Given that dairy products are typically fortified with vitamin D, a negative association between dairy intake and MS risk might be expected. However, work utilizing the Nurses’ Health Study cohorts showed an increased risk for developing MS among women with high intake of whole milk during adolescence [88]. Women who consumed whole milk 3 or more times per day had a 47% increased risk of developing MS compared to those who consumed < 1 serving per day. Several hypothetical mechanisms may explain this finding. MS patients have showed abnormally heightened T cell responses to milk antigens, differing at particular epitopes from patients with type 1 diabetes and those without autoimmune disease in both children [89] and adults [90]. The milk protein butyrophilin has been implicated through antigenic mimicry with myelin oligodendrocyte glycoprotein in EAE [91] as well as in MS patients [92]. Gut microbiota may also contribute to these effects. For example, in an animal model of inflammatory bowel disease, a high milk fat diet was associated with increased incidence and severity of colitis, linked to the proliferation of *Bilophila wadsworthia* and induction of a T_H_1 immune response [93].

Within the HOLISM (Health Outcomes in a Sample of people with MS) study, 2047 patients with confirmed MS completed a dietary questionnaire in addition to providing information on MS status [94•]. Participants who reported not consuming dairy were less likely to report recent disease activity and reported higher health-related quality of life compared to those who reported consuming dairy. In contrast, a study utilizing the North American Research Committee on MS (NARCOMS) registry noted an inverse relationship between total dairy intake and disability severity (top vs. bottom quintile, for severe vs. mild disability OR = 0.77, *p* = 0.009) [95••]. Notably, these studies were not able to distinguish between different types of dairy.

### Fruits, Vegetables, and Whole Grains

In the HOLISM study described above, higher intake of fruits and vegetables was associated with reduced levels of patient-reported disease activity and disability [94•]. The prospective pediatric MS study discussed above that noted an increased risk of relapse relating to increased saturated fat intake also noted a reduction in relapse rates with increasing intake of vegetables [68••]. Excluding potatoes and legumes, a one-cup equivalent increase in vegetable intake decreased the risk of relapse by 50% (hazard ratio 0.50, *p* = 0.024).

A pilot study evaluated effects of a high vegetable/low protein diet (HV/LP, *n* = 10) compared to a typical “Western diet” (WD, *n* = 10) in MS patients for 12 months [96]. As compared with WD, the HV/LP group showed a decrease in pro-inflammatory IL-17+ and PD-1+ T cells and an increase in anti-inflammatory PD-L1+ monocytes. In analyses of gut microbiota, the family *Lachnospiraceae* was noted to be more abundant in the HV/LP group compared to the WD group. Within the HV/LP group only, *Lachnospiraceae* abundance was correlated with (anti-inflammatory) IL-10+ and TGFβ+ monocytes and Treg cells.

Little work has been completed with relation to whole grains; however, they are included here because a recent study suggests an association with MS disease course and there is a potential mechanistic connection that also applies to fruits and vegetables that are high in fiber. The NARCOMS study described above noted an inverse relationship between whole grain intake and MS-related disability [95••]. Participants in the top vs. bottom quintile of whole grain consumption had lower odds of severe vs. mild disability (odds ratio 0.78, *p* = 0.02).

A mechanistic link may lie in the capacity for gut microbiota to ferment high fiber foods (certain grains, vegetables, fruits) to short-chain fatty acids (SCFAs). SCFAs favor immunomodulation by promoting intestinal epithelial cell integrity, inducing the differentiation of Tregs [64], and reducing the production of pro-inflammatory cytokines and chemokines, among other effects, mediated through activation of GPCRs and HDAC inhibition [97]. In EAE, pretreatment with the SCFA propionic acid (PA) ameliorated the disease course, with observed increase in production of the anti-inflammatory cytokine IL-10 and in the number of Foxp3 + Tregs [63]. Interestingly, the transfer of Tregs from PA-treated mice resulted in improved clinical EAE course and histological markers of EAE severity compared to transfer of similar number of Tregs from untreated mice, consistent with important effects of PA on Treg function as well as number.

Other mechanisms may explain the potential link between the intake of fruits and vegetables and MS. For example, cruciferous vegetables provide the essential amino acid tryptophan. Tryptophan’s metabolites, generated by the diet directly as well as by gut microbiota, activate the aryl hydrocarbon receptor (AhR). Through AhR, these metabolites have multiple actions relevant to the peripheral immune system including induction of FoxP3+ Tregs (direct effects on transcription and indirect effects through dendritic cell modulation) and IL-10-producing type 1 regulatory T cells (Tr1) as well as interference with T_H_17 cell differentiation [41]. In addition, several of these metabolites are able to cross the blood-brain barrier and activate AhR on astrocytes [43••]. AhR interaction with SOCS2 inhibits NF-κB, ultimately inhibiting local monocyte recruitment and activation, microglial activation, and neurotoxicity [42, 43••]. These effects on the local CNS environment make further investigation of this pathway, particularly regarding the elusive neurodegenerative component of MS, highly significant.

Flavonoids, phytopigments found in fruits and vegetables (and other foods such as coffee and tea) representing the most abundant class of polyphenols, are also AhR agonists [98]. They act through a variety of other mechanisms relevant to immunomodulation and neurodegeneration as well [99, 100••]. Studies of many different flavonoid compounds have demonstrated effectiveness in MS animal models including attenuation of EAE [101, 102], neuroprotection [103, 104], and even promotion of remyelination [105, 106]. However, it is important to note that studies in MS models have largely related to flavonoid compounds rather than foods. Positive effects of foods and food extracts such as strawberries, spinach, and blueberries have been demonstrated in other neurodegenerative diseases [107, 108], and one study of freeze-dried blueberries in EAE showed a protective effect [109].

### Salt

Preclinical studies have suggested potential adverse effects of a high salt diet in MS. Pro-inflammatory T_H_17 cell differentiation is induced by high salt intake modulated through serum glucocorticoid kinase 1 (SGK1) [110]. T_H_17 cells that develop in a high salt environment demonstrate a more pathogenic phenotype and mice fed a high salt diet exhibit worsened course of EAE [111]. Translating this work into humans, investigators estimated dietary sodium intake in 70 relapsing remitting MS patients utilizing a single spot urine sample and then followed them for 2 years [112]. Compared to those in the low sodium intake group, those with medium or high intake had clinical relapse rates 2.75- and 3.95-fold higher, respectively. Similar results were noted in a validation cohort of 52 patients.

However, additional studies have not confirmed these findings. A case-control study in pediatric MS using a food frequency questionnaire to estimate dietary sodium intake found no association with MS risk among 170 MS cases and 331 controls [113]. An observational study utilizing the same pediatric MS network found no association between sodium intake and risk of relapse among 174 relapsing remitting MS patients followed for a median of 1.8 years [114]. An additional study in adults utilized stored 24 h urine samples from the BENEFIT (Betaferon/Betaseron in Newly Emerging Multiple Sclerosis for Initial Treatment) trial in which over 400 patients with an initial MS relapse were randomized to be treated with interferon or placebo for 2 years followed by a 3-year extension phase resulting in a cohort with approximately 5 years of follow-up [115]. Patients had a median of 14 urine samples collected over the study period from which dietary sodium could be estimated. There was no association between estimated dietary sodium intake and relapse rates or MRI outcomes in this cohort.

There are currently no published clinical trials specifically aimed at reduction of sodium intake in MS. A study examining effects of high vs. low salt diet on T_H_17 and regulatory T cells in MS patients and healthy controls is currently recruiting (NCT02282878).

## Dietary Patterns in Multiple Sclerosis

In considering the effects of macro or micronutrients and singular foods or food groups, it is important to note that these individual components are not ingested in isolation. Rather, the diet is comprised of many components ingested together as part of an overall program and there are likely significant interactions. Therefore, examination of the effects of global dietary patterns is extremely important.

### Overall Dietary Quality

Two studies have leveraged existing MS registries to evaluate potential associations between overall diet quality and MS-related disability and symptomatology. Within the HOLISM (Health Outcomes in a Sample of people with MS) study, as described above, 2047 patients with confirmed MS completed the Diet Habits Questionnaire (DHQ) as part of a comprehensive survey including information on relapse rate, disability status, and quality of life [94•]. The study noted that every 10-point increase on the DHQ (overall score ranging from 0 to 50, higher scores indicating higher quality diet) was associated with a 30% less likelihood of higher disability level. Higher DHQ scores were also significantly associated with better physical and mental health-related quality of life (HRQOL). Similarly, 6989 participants in the North American Research Committee on MS (NARCOMS) Registry, as described above, completed a dietary screener questionnaire (DSQ) in addition to providing information on recent relapses, progression, and disability [95••]. Participants in the top quintile of diet quality score were at 20% lower odds of higher disability scores compared to those in the bottom quintile. Higher diet quality was also linked to decreased odds of more severe depressive symptoms, after adjusting for disability status.

### Caloric Restriction

Caloric restriction has gained attention in diverse fields in medicine, particularly in aging [116]. Observations in the aging literature including increase in endogenous corticosteroid production, decrease in inflammatory cytokines, and increase in neurotrophic factors led to initial experiments demonstrating that rats fed a severely calorie-restricted diet (66% food restriction) were protected from the development of EAE [117, 118]. Further experiments with 40% calorie restricted diet in mice showed decreased EAE severity as well as reduced inflammation, demyelination, and axonal damage [119].

There are obvious challenges to translating significant long-term caloric restriction to humans. A potential solution is the use of intermittent caloric restriction, which has also been demonstrated as beneficial in EAE [120, 121]. Mice fed three cycles of a “fasting mimicking” diet (FMD; very low calorie diet lasting 3 days every 7 days) exhibited delayed EAE onset, reduced EAE incidence, and decreased EAE severity [121]. These clinical findings were accompanied by reduced immune cell infiltration and demyelination in the spinal cord on histology. Lymphocytes isolated from draining lymph nodes and spleens showed decreases in T_H_1 and T_H_17 cells and increase in Treg cells in mice fed FMD compared to controls. FMD also protected oligodendrocytes from apoptosis and stimulated oligodendrocyte regeneration and differentiation suggesting an important impact on neuroprotection and repair. These effects were confirmed to be present independent of inflammation, demonstrated through use of a cuprizone model (toxic rather than inflammatory demyelination model).

Toward translating this work to MS patients, a pilot clinical trial randomized 60 relapsing remitting MS patients to a control diet for 6 months, ketogenic diet (KD) for 6 months, or single cycle of modified FMD for 7 days followed by Mediterranean diet for 6 months [121]. The diets were well tolerated, and participants adhered to them well. Participants assigned to FMD and KD had higher health-related quality of life scores at 3 months compared to controls.

A current pilot study is evaluating feasibility and effects of continuous and intermittent caloric restriction compared to controls for approximately 1 year in MS patients (NCT02647502).

### McDougall Diet

The McDougall Diet is a very low-fat (10% of calories from fat) diet consisting mostly of starchy plant-based foods as well as other vegetables and fruits. No animal products or oils are permitted. In a recent study, 61 participants with relapsing remitting MS were randomized to follow the McDougall diet or participate as a wait-list control for 12 months [122]. The primary end point, the number of new T2 lesions on MRI, was not satisfied; however, notably the study was powered to detect only a very large effect. There were no differences in clinical relapse rates. There was a significant impact on fatigue, though much of the effect was attributable to weight loss. A larger randomized trial of this diet for fatigue is currently underway (NCT03322982).

### Paleolithic Diet

Paleolithic diets have become popular in recent years, both for the general population and among people with MS. While there is some variability regarding allowable foods, Paleolithic diets generally emphasize consumption of lean meats including organ meats, fish, vegetables, and fruits and typically do not permit consumption of dairy or grain products. Despite much attention in the lay literature, to date, there are few scientific studies incorporating a Paleolithic diet for MS. A multimodal intervention including a program of a modified Paleolithic diet, dietary supplements, and a physical exercise program along with electrical stimulation and meditation significantly improved fatigue in patients with secondary progressive MS [123]. This was an open label pilot study that enrolled 10 subjects, only 6 of whom completed the study with good adherence. Additional studies are currently underway (NCT02687919, NCT02914964).

### Mediterranean Diet

There has been little work on Mediterranean-style diets in MS. However, the preliminary evidence reviewed above regarding the role of various dietary components suggests that this type of diet may be of benefit. Mediterranean-style diets are low in saturated fats, high in polyunsaturated and monounsaturated fats (especially fish and olive oil), high in fruits and vegetables, and low in processed foods implying low salt content. Additional support for further study of this pattern in MS comes from the aging literature, where level of adherence to Mediterranean diet has been associated with structural measures of neurodegeneration [85•] and the presence of Alzheimer’s disease or mild cognitive impairment [124], as well as related to degree of cognitive decline longitudinally [124]. A pilot clinical trial of a modified Mediterranean diet in MS is currently underway (NCT02986893).

## Limitations of Current Dietary Research in Multiple Sclerosis and Future Directions

Research regarding the role of diet in multiple sclerosis is advancing but currently remains limited. Few studies have been prospective with rigorously collected outcomes, and the few clinical trials that have been conducted have not been of sufficient size or length to adequately assess efficacy. Moving this field of research forward presents several challenges. Further work needs to be done regarding assessment tools for recording dietary habits in MS patients. Epidemiologic and observational studies of diet are confounded by other behaviors such as smoking and exercise. These can be accounted for in statistical models, but these methods are imperfect and the possibility of unmeasured confounders always exists.

Regarding clinical trials, the goal is to hold their design to the same rigorous standards applied to pharmaceuticals. However, this is rather difficult. Participants must be willing to volunteer to be randomized; MS patients who are willing to agree to potentially make a big dietary change may be disappointed by assignment to a control group which may result in dropouts and crossovers. Given the nature of the intervention, blinding of participants is not possible. Appropriate end points present another challenge. For example, patients with relapsing MS are likely to be on effective MS disease-modifying immunomodulatory therapies, reducing the ability to detect effects of diet on relapse rates and new MRI lesions. The research community is still addressing optimal end points to be used in trials of progressive MS.

## Conclusion

Multiple sclerosis incidence and disease course are clearly influenced by environmental factors. While some factors are well described, the contribution of other mediators has not been fully elucidated. The initial epidemiologic investigations regarding a potential role for diet in MS date back many years. More recently, preclinical models, epidemiologic research, a small number of prospective studies, and limited clinical trials suggest the importance of various dietary factors in MS. Mechanistic experiments highlight potential effects of diet on both immunomodulatory and neurodegenerative processes in MS. Further research on this topic, ranging from continued basic science studies to clinical trials, is currently ongoing.
